# A Simple Approach for Synthesizing of Fluorescent Carbon Quantum Dots from Tofu Wastewater

**DOI:** 10.1186/s11671-017-2369-1

**Published:** 2017-11-29

**Authors:** Jin Zhang, Hong Wang, Yiming Xiao, Ju Tang, Changneng Liang, Fengyan Li, Haiming Dong, Wen Xu

**Affiliations:** 1 School of Physics and Astronomy and International Joint Research Center for Optoelectronic and Energy Materials, Yunnan University, Kunming, 650091 People’s Republic of China; 2 Key Laboratory of Materials Physics, Institute of Solid State Physics, Chinese Academy of Sciences, Hefei, 230031 People’s Republic of China; 3 Department of Physics, China University of Mining and Technology, Xuzhou, 221116 People’s Republic of China

**Keywords:** Fluorescence, Carbon quantum dots, Tofu wastewater, Hydrothermal reaction

## Abstract

We present an investigation on carbon quantum dots (CQDs) synthesized from wastewater induced during the production of tofu. We find that tofu wastewater is a good source of raw material in making fluorescent CQDs. The corresponding CQDs can be fabricated simply via hydrothermal reaction to carbonize the organic matter in the yellow serofluid of tofu wastewater. Two sorts of CQDs can be obtained within the deionized water and NaOH solution, respectively, where the CQDs in water (NaOH solution) can emit blue (green) light under the UV irradiation. It is found from X-ray photoelectron spectroscopy (XPS) that the basic difference between these two sorts of CQDs is the contents of C–O and C=O bonds on the surface of the CQDs. This difference can cause different features of the photoluminescence (PL) spectra of the CQDs. On the basis of the obtained results from the XPS and PL measurements, we propose a mechanism in understanding and explaining the photon-induced light emission from CQDs. This study is relevant to the fabrication and application of fluorescent CQDs as, e.g., light display materials.

## Background

Tofu, made from soybean, is the daily food in China and in the Asian community. In the past, tofu and related products were mainly made by families and small factories in relatively small amount. With the vegetarian being more and more popular worldwide, the demand of tofu products has been rapidly increasing in the last two decades since the big international supermarkets such as WalMart and Carrefour sold these as health-foods. Nowadays, tofu and related products are mainly mass-produced by big factories in the industrial park in China. However, one of the environmental issues of mass production of tofu in the industrial park is the wastewater. The production of soybean products would result in wastewater mixed with soybean yellow serofluid. This wastewater can cause environmental pollution. On the other hand, tofu yellow serofluid is highly concentrated with organic matters and contains carbohydrates, proteins, organic acids, functional oligosaccharides, water-soluble non-protein nitrogen and vitamins, lipids, and other pigment substances. Therefore, it is a good source of raw material in fabricating carbon quantum dots (CQDs) for optics, biomedicine, and other applications. Thus, applying tofu wastewater to make CQDs can reuse the wastes from mass production of tofu and largely reduce environmental pollution. These become the prime motivation of our present study.

Carbon quantum dots is a new class of carbon-based nanomaterial normally with spatial size of 20 nm or less [1, 2]. It has been found that the CQDs are of good water solubility, high chemical inertness, low toxicity, and excellent biocompatibility [3, 4]. From a viewpoint of physics, the electronic energy spectrum for a CQD is akin to a direct band-gap semiconductor. Thus, the CQDs have been proposed as fluorescent materials for advanced optical and optoelectronic devices [5, 6]. In recent years, the CQDs have been rather intensively investigated. A variety of fabrication methods and different sources of raw materials have been applied to realize the CQDs for optical applications [5–7]. In general, the synthesis of CQDs can be achieved through top-down and bottom-up approaches [8]. The top-down method is mainly a physical approach in forming carbon dots by breaking or peeling larger carbon material structures, including arc discharge [9], electrochemical oxidation [10], chemical oxidation [11], laser ablation [12], etc. The bottom-up method is to employ the small molecules as precursors to obtain CQDs through chemical reactions, including combustion [13], microwave [14], and ultrasonic [15] approaches along with chemical solution synthesis [16], hydrothermal reaction [17], etc.

In recent years, biomass such as wheat straw [18] and plant leaves [19] has been widely used as carbon sources for synthesis of the CQDs. Moreover, water-soluble fluorescent CQDs have been prepared by hydrothermal treatments of orange juice [20] and Jinhua bergamot [21] which are taken as carbon sources. Such a simple approach has been applied for the large-scale synthesis of water-soluble CQDs from many sorts of food waste-derived sources [22].

In this study, we take tofu yellow serofluid as carbon source to synthesize the CQDs via employing the hydrothermal method to carbonize the organic matters in the yellow serofluid. It has been pointed out [17] that the hydrothermal method is an easy and low-cost approach which can be applied to large-scale and one-step synthesis of water-soluble fluorescent CQDs. For optical application of the CQDs, especially as light display materials, it is desirable to be able to produce the fluorescent CQDs which can emit blue, green, and red radiation. Our current research work is being conducted along this direction. In the present study, we prepare a series of fluorescent CQDs for investigation. The transmission electron microscopy and the X-ray photoelectron spectroscopy are applied for the characterization of the fabricated CQDs. The photoluminescence experiment is employed to measure the optical properties of the CQDs.

## Methods

In this study, the wastewater from tofu production is taken from the Tofu Industrial Park in Shi Ping County, Yunnan, China. The general processes to synthesize the CQDs from yellow serofluid in tofu wastewater can be described as follows: (i) We prepare the carbon precursory materials via pyrolysis of the tofu yellow pulp in wastewater. Here, 300 ml of tofu yellow syrup is put into the 500-ml beaker and placed onto the heating platform for constant heating. We find that when heating temperature is at about 93 °C and the heating time is for 3 to 5 h, the tofu yellow serofluid in the beaker can become burning dry. (ii) We let the stuff in the beaker cool down naturally till room temperature and add 50–200 ml deionized water into the beaker. (iii) The mixture is magnetically stirred for 4 min to achieve the uniform and full mix of the matters and water. (iv) The mixture is taken for 5 min ultrasonic shock to break the loosing clusters. Thus, we can obtain the supernatant which contains carbon dots. (v) The supernatant is further centrifuged at a speed of 12,000 r/min for 20 min, and the further supernatant can be obtained. As a result, the CQDs can be finally acquired within deionized water. It is found that the heating temperature, the heating time, and the pH value of the yellow pulp water in the synthesis process can affect rather strongly the growth of the CQDs. Therefore, the CQDs can be fabricated with certain fluorescent features through varying the above synthesis conditions. We notice from bare eye observation with daylight that the supernatant with CQDs prepared under abovementioned experimental conditions looks yellow. However, it can look blue under the UV irradiation. We name this sort of fluorescent CQDs as CQDs-1 in this article.

By taking the similar synthesis approach, we can produce the CQDs via using NaOH as solution for burning dry tofu yellow serofluid after pyrolysizing, instead of using deionized water discussed above. We add 100 ml of NaOH solution with a pH value about 12.4. Following the same processes of magnetic stirring, ultrasonic shocking, and centrifuging as stated above, we can also acquire the CQDs within the NaOH solution. These CQDs also look yellow from bare eye observation with daylight. However, they can look green under the UV irradiation. We name this sort of fluorescent CQDs as CQDs-2 in this article.

In this work, we have made two types of CQDs which can emit green and blue lights under the UV irradiation. The further investigation of the present work is conducted mainly for these two types of CQDs realized from tofu wastewater.

## Results and Discussions

**Fig. 1:**
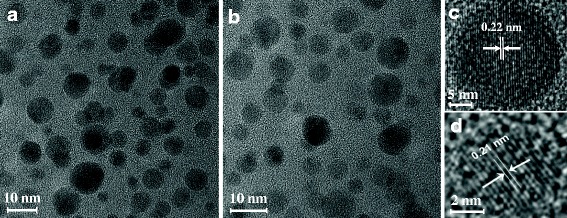
**a** TEM images for CQDs in deionized water (CQDs-1) and **b** TEM images for CQDs in NaOH solution (CQDs-2). **c**, **d** Zoomed-in image of a single CQD of **a** and **b**, respectively

For characterization of the CQDs synthesized from tofu wastewater, we first carry out the morphological analysis for these CQDs. In Fig. 1, we show the typical image of the CQDs within deionized water and NaOH solution (CQDs-1 and CQDs-2), obtained from high-resolution transmission electron microscopy (TEM). As we can see, the prepared CQDs are spherical and mono-dispersive within the deionized water (for CQDs-1) or NaOH solution (for CQDs-2). Through a statistical average of the TEM image, the particle size of these CQDs is in the range from 2 to 10 nm. We find that these CQDs are highly crystallized with typical lattice structure of carbon. The lattice fringes are clear and the corresponding lattice spacing is about 0.22 and 0.21 nm, respectively. We would like to note that the results shown in Fig. 1 are very similar to those reported previously for the N- and S-doping content in N- and S-CQDs with high yield [23, 24]. Moreover, we find that the size distribution of the CQDs in deionized water (CQDs-1) or in NaOH solution (CQDs-2) is mainly located around 3.5–5.5 nm and the thickness of these CQDs is about 3.5 nm.

**Fig. 2:**
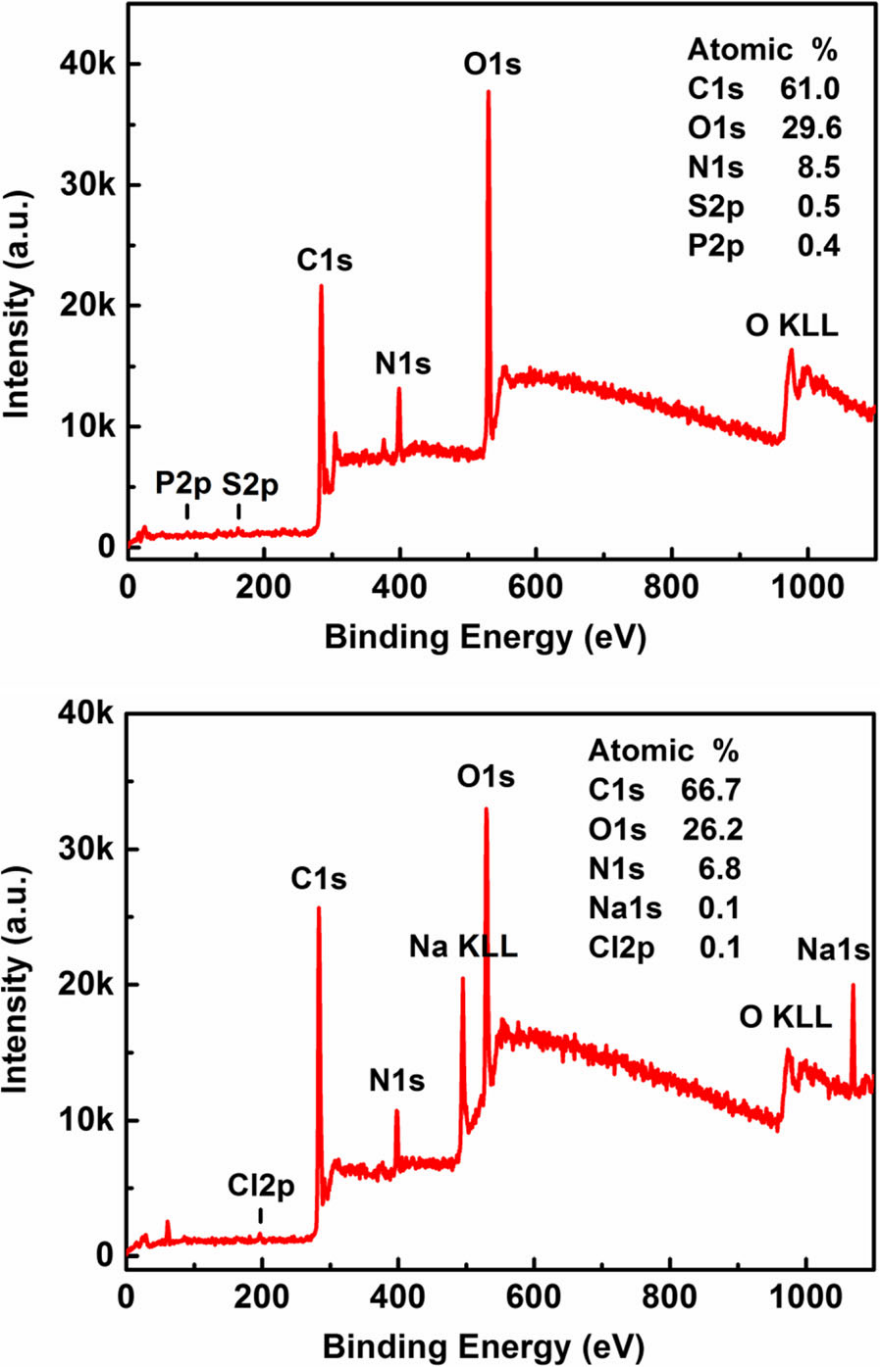
The XPS full spectrum for CQDs-1 (upper panel) and CQDs-2 (lower panel), respectively, where the obtained contents of elements are indicated

As we know, the X-ray photoelectron spectroscopy (XPS) is a powerful tool for the measurement and understanding of the elemental compositions and the content of the CQDs, especially for the examination of surface-modified features of the CQDs such as the functional groups on the surface of the CQDs [25]. In Fig. 2, the XPS full spectra for CQDs-1 and CQDs-2 are presented and the corresponding findings are indicated. We notice that the CQDs measured here contain mainly C (with a typical binding energy C ls = 284.8 eV), N (with a typical binding energy N ls = 400 eV), and O (with a typical binding energy O ls = 532 eV). The other elements such as S and P (Na and Cl) can also be found in CQDs-1 (CQDs-2). As a result, we see that CQDs-1 is mainly composed of C, N, O, S, and P elements, in which the atomic ratio of these elements is C1s:O1s:N1s:S2p:P2p = 61.0:29.6:8.5:0.5:0.4. We also see that CQDs-2 is mainly composed of C, O, N, Na, and Cl elements. The atomic ratio of these elements is C1s:O1s:N1s:Na1s:Cl2p = 66.7:26.2:6.8:0.1:0.1. Because the tofu wastewater itself contains chloride and sulfate induced by the process in making tofu, there are rather broad spectra of S and Cl signals in Fig. 2. Moreover, because CQDs-2 is for CQDs in NaOH solution in which NaOH can play a role as passivation of the CQDs, there is a Na signal in the lower panel of Fig. 2.

**Fig. 3:**
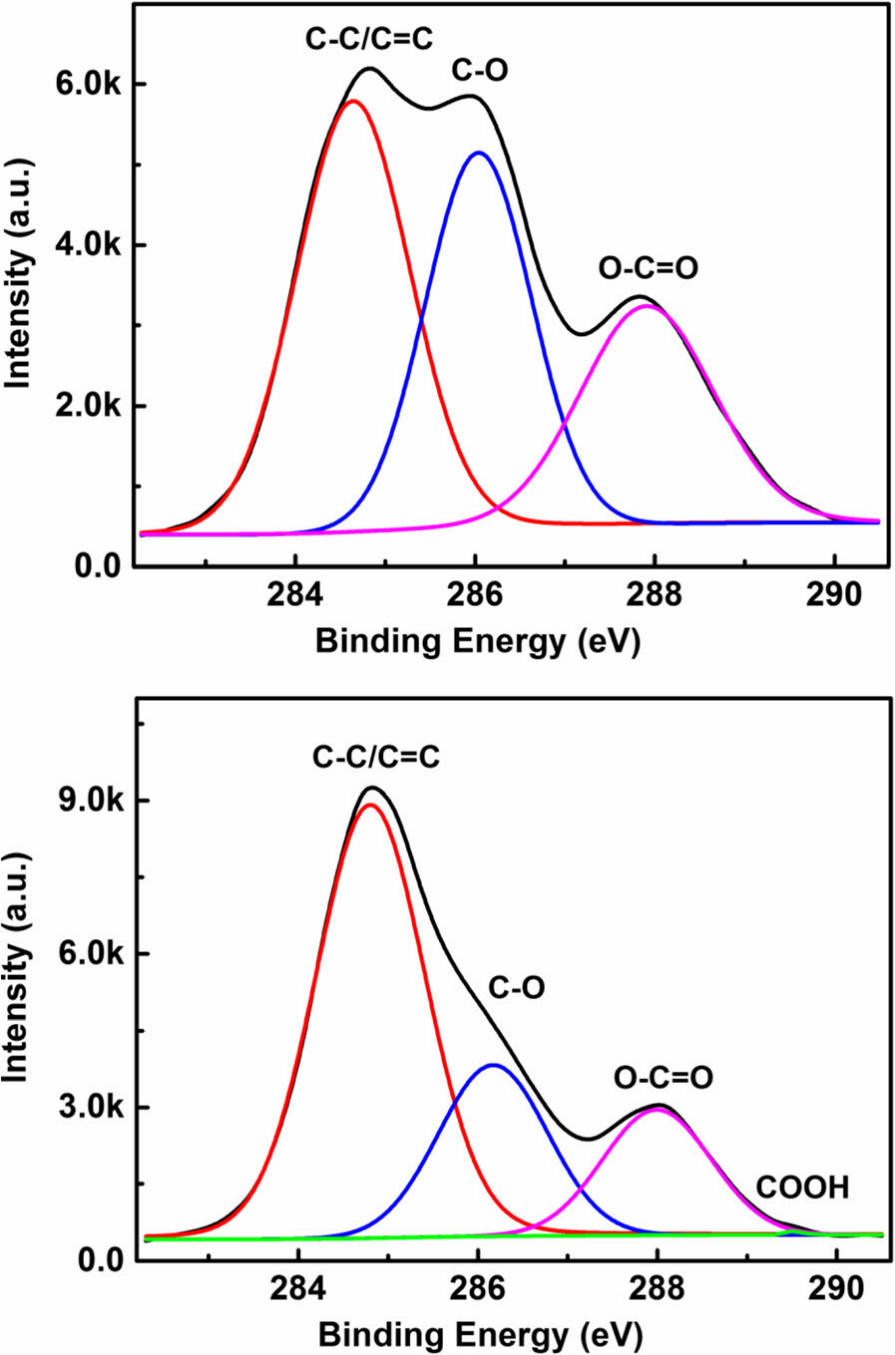
The high resolution C1s spectrum for CQDs-1 (upper panel) and CQDs-2 (lower panel), respectively, fitted by a binding energy C1s

In Fig. 3, we show the high resolution C1s spectra for CQDs-1 and CQDs-2, respectively, fitted by a binding energy Cls. It can be seen from the C1s spectrum in the upper panel of Fig. 3 that three chemical bonds C–C/C=C at 284.7 eV, C–O at 286.08 eV, and C=O at 287.86 eV present in CQDs-1. There are four chemical bonds C–C at 284.8 eV, C–O at 286.16 eV, C=O at 288 eV, and COOH at 289.14 eV present in CQDs-2, as shown in the lower panel of Fig. 3. From the XPS results shown in Fig. 3, we learn that the basic difference between CQDs-1 and CQDs-2 is the contents of C–O and C=O bonds on the surface of the CQDs within water and NaOH solution, respectively. It is known that the OH^−^ in NaOH solution can couple with C–O and C=O bonds on the surface of the CQDs to form COOH and the carboxyl group and, thus, to reduce the contents of the C–O and C=O groups in CQDs-2. This is the main reason why the contents of C–O and C=O bonds in CQDs-1 are markedly higher than those in CQDs-2.

**Fig. 4:**
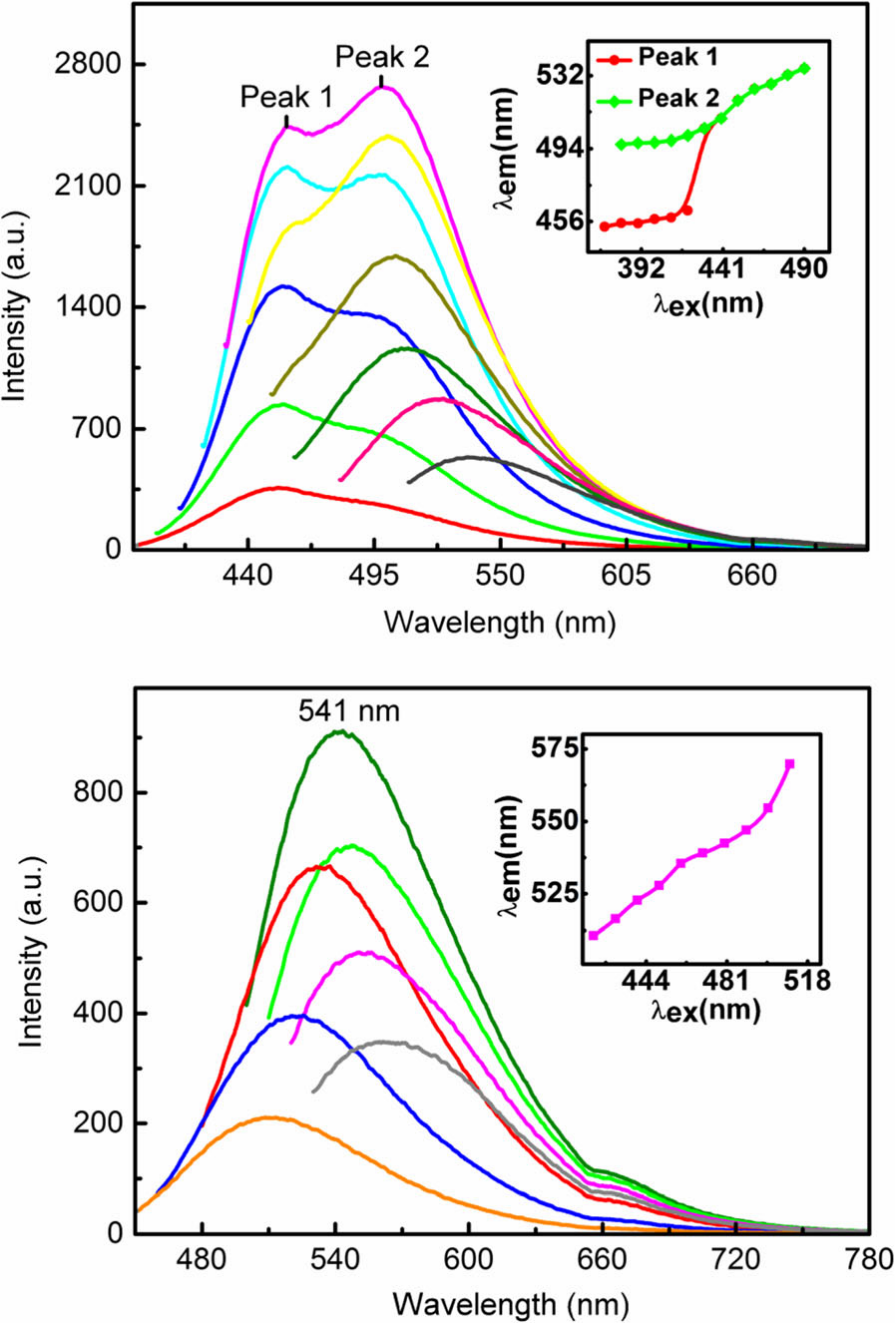
The PL spectrum for CQDs-1 in upper panel and CQDs-2 in lower panel at different excitation wavelengths *λ*_ex_. In the upper panel, *λ*_ex_ are 370 nm (red), 380 nm (green), 390 nm (blue), 400 nm (light blue), 410 nm (deep pink), 420 nm (yellow), 430 nm (light green), 440 nm (dark green), 450 nm (light red), and 490 nm (dark olive green). In the lower panel, *λ*_ex_ are 420 nm (orange), 440 nm (blue), 460 nm (yellow), 480 nm (red), 490 nm (green), 500 nm (pink), and 510 nm (dark olive green). The inserts show the peak wavelength in the PL spectrum, *λ*em, as the function of excitation wavelength

In this study, we take a standard experimental setup to measure the photoluminescence (PL) emission from CQDs realized from tofu wastewater in visible bandwidth. The HORIBA fluorescence system (USA) is applied for the measurement, where a xenon lamp is taken as broadband excitation light source, the GEMIMI 180 mono-chromator is used for choosing the optical pumping wavelength, and the iHR320 grating spectrometer together with a photoelectric multiplier tube (PMT) detector is used for recording the spectrum of the light emission from samples. The measurements are carried out at room-temperature. In Fig. 4, we show the PL emission spectra for CQDs-1 in the upper panel and CQDs-2 in the lower panel at different excitation wavelengths *λ*_ex_. For the PL measurement, the recording of the intensity of the emission light often starts after the excitation wavelength to void the damage of the PMT detector. Thus, there have been cutoffs in the curves of the PL spectra in Fig. 4. We notice the following features: (i) The intensity of the PL emission first increases then decreases with increasing excitation wavelength. The strongest PL emission can be observed at about *λ*_ex_ ~ 410 nm for CQDs-1 and 480 nm for CQDs-2, respectively. (ii) The peak wavelength position *λ*_em_ in the PL spectrum varies with altering the excitation wavelength for both CQDs-1 and CQDs-2. In the inserts of Fig. 4, we show *λ*_em_ as a function of *λ*_ex_ so we can see more clearly how the PL peaks shift with excitation wavelength. As shown in Fig. 4, *λ*_em_ increases monotonously with *λ*_ex_ for both CQDs-1 and CQDs-2. (iii) In relatively shorter excitation wavelength regime, two PL peaks can be observed for CQDs-1, whereas only one PL peak can be seen for CQDs-2 over the 420–510 nm wavelength regime. (iv) CQDs-1 can result in a more broadened PL spectrum than CQDs-2 can. (v) The PL peak wavelength induced by CQDs-1 is shorter than that induced by CQDs-2. At 410 nm excitation wavelength, the blue fluorescence can be achieved by CQDs-1, whereas at 480 nm excitation wavelength, the green fluorescence can be seen for CQDs-2. (vi) The fluorescence of the CQDs-1 with 8.5% N-doping content is higher than that of the CQDs-2 with 6.8% N-doping content. The reason why the PL emission increases with N-doping content of CQDs is that N-doping can introduce a new kind of surface state. Electrons trapped by the new formed surface states are able to facilitate a high yield of radiation recombination [24]. The PL results obtained from this study indicate that the blue and green light emission can be achieved by CQDs-1 and CQDs-2, respectively, under optical pumping.

At present, the physical mechanism for photon-induced light emission from CQDs is still unclear. However, the results obtained from related investigations [12, 26, 27] have shown that the surface modification of the CQDs by amino and carboxyl functional groups can play an important role for the PL emission from CQDs. The features of the PL spectrum of CQDs are determined not only by the particle size of the CQDs [1] but also by the surface properties of CQDs [26, 27]. Based on our XPS and PL results obtained from the present study, we now discuss the physical mechanism behind the experimental findings shown in Fig. 4 for CQDs realized from tofu wastewater. We know that the electronic band structure of CQDs is very much similar to that in a direct band gap semiconductor. However, for CQDs synthesized from tofu wastewater in different solutions such as water and NaOH, there are C–O, C = O, and COOH bond-based functional groups on the surface of the CQDs, as shown by the XPS results in Fig. 3. The energy states of these functional groups are surface states which are located in between the conduction and valence bands of the CQDs. They play a role like intermediate states, very similar to impurity states in a direct band gap semiconductor. In the presence of excitation light field, the electrons in the valence band of the CQDs are pumped into the conduction band via optical absorption mechanism. Because the position of the PL peak in the spectrum depends on the excitation wavelength, the PL emission via excitonic mechanism [28] is not the case for these CQDs. The photon-induced light emission from CQDs is therefore a consequence of the direct photoemission induced by electronic transitions from higher energy levels to lower energy states. As we know, the electrons are normally with a quicker or smaller relaxation time in the higher energy states than that in the lower energy states. The results from our XPS and PL measurements suggest that the radiative electronic transition in CQDs is mainly achieved via relaxation of electrons from the surface states to the valence band of the CQDs. The obtained experimental results show that the intensity of the PL emission from CQDs prepared by KOH is much stronger than that prepared by NaOH. With the same excitation wavelength, we find that the alkali ion in alkaline solutions do not affect significantly the position of the PL emission wavelength.

For the case where the CQDs are in water (CDQs-1), there are two intermediate states induced by surface states of the C–O and C=O bonds and related functional groups. These two surface states are with different energy levels and corresponding selection rules for radiative electronic transitions, which are responsible for the emission of PL with two emission wavelengths under relatively short wavelength light excitation. The photoexcited electrons in the higher energy states in the conduction band of CQDs first quickly relax into the surface states via non-radiative relaxation mechanism such as electron-phonon scattering and electron-electron interaction. When the non-radiative electronic relaxation time for electrons in the surface states is longer or larger than the radiative electronic relaxation time, these electrons can go back to the valence band and emit photons. With decreasing pumping wavelength, more states in the valence band and especially in the conduction band can take part in this pumping, relaxation, and light emission process and, thus, the peak wavelength in the light emission spectrum decreases with excitation wavelength. Therefore, the wavelength of the light emission depends on the excitation light wavelength. The increase in the peak wavelength of light emission with excitation wavelength implies that the non-radiative electronic relaxation time increases with lowering energy levels in the surface states. For relatively long wavelength light excitation, the photoexcited electrons in CQDs are quickly relaxed from the conduction band to the lower energy levels of the surface states and emit photons. The possibility for the emission of photons from higher energy levels of the surface states becomes low enough so that the effect cannot be markedly measured.

For the case where the CQDs are in NaOH solution (CDQs-2), there is only one intermediate state for the radiative electronic transitions. Because the contents of the C–O and C=O bonds and related functional group are relatively low in this case, the radiative surface states are mainly induced COOH based groups for CQDs-2. As a result, only one peak of the PL emission can be observed. Since the energy levels of the surface states induced by C–O and C=O bonds and related functional groups are normally higher than those induced by COOH groups, the shorter wavelength PL emission can be observed for CQDs-1. This is the main reason why CQDs-1 can emit blue light whereas CQDs-2 can emit green light under optical excitation.

The quantum efficiency *Q* of the fluorescence for CQDs-1 can be evaluated from the experimental data via [29, 30]

1 $$ Q={Q}_{\mathrm{s}}\times \frac{I_{\mathrm{s}}}{I}\times \frac{A}{A_{\mathrm{s}}}\times \frac{\eta^2}{{\eta_{\mathrm{s}}}^2} $$

Here *Q*_s_ is the quantum efficiency of the fluorescence for a standard sample for reference. Under a fixed excitation wavelength at, e.g., 364 nm, *I* and *I*_s_ are the integrated emission intensities of the CQDs-1 sample and the standard sample, respectively. *A* and *A*_s_ are respectively the absorbance of the prepared sample and standard sample at the same excitation wavelength. *η* and *η*_s_ are respectively the refractivity of the prepared sample and standard sample. It is found that the fluorescent quantum efficiency of CQDs-1 is about 54.49%. Because we cannot find the reference sample for CQDs-2, the fluorescent quantum efficiency of CQDs-2 is not evaluated in the present study.

## Conclusions

In this study, we have fabricated the carbon quantum dots (CQDs) from wastewater induced during the production of tofu. We have demonstrated that the tofu wastewater is a good source of raw material in making CQDs. The fluorescent CQDs can be fabricated simply via hydrothermal reaction to carbonize the organic matters in the yellow serofluid of tofu wastewater. The average size of the CQDs synthesized from tofu wastewater can be up to 3.5 nm. We have obtained two sorts of CQDs within the deionized water and NaOH solution, respectively. They can emit blue and green lights, respectively, under the UV irradiation. It is found from the X-ray photoelectron spectroscopy (XPS) that the basic difference between these two sorts of CQDs is the contents of C–O and C=O bonds on the surface of the CQDs. This difference can cause different features of photoluminescence (PL) spectrum of the CQDs. On the basis of the obtained results from the XPS and PL measurements, we have proposed a mechanism in understanding and explaining the photon-induced light emission from CQDs. One of the most significant conclusions obtained from this study is that using tofu wastewater to synthesize the CQDs can be not only helpful to provide a solution of the environmental problem caused by the wastewater but also promising for simple and low-cost mass production of CQDs for bio- and optical applications. We have so far successfully obtained the blue and green fluorescent CQDs from tofu wastewater. The challenge of our current work is to obtain the CQDs which can emit red light under optical pumping.
